# Assessment of a recombinant protein from *Leishmania infantum* as a novel tool for Visceral Leishmaniasis (VL) diagnosis in VL/HIV co-infection cases

**DOI:** 10.1371/journal.pone.0251861

**Published:** 2021-05-17

**Authors:** Rhaíssa E. M. Ramos, Wagner J. T. Santos, Franklin B. Magalhães, George T. N. Diniz, Carlos H. N. Costa, Osvaldo P. de Melo Neto, Zulma M. Medeiros, Christian R. S. Reis

**Affiliations:** 1 Instituto Aggeu Magalhães, Fundação Oswaldo Cruz, Recife, Pernambuco, Brazil; 2 Centro Universitário Tabosa de Almeida, Caruaru, Pernambuco, Brazil; 3 Instituto de Doenças Tropicais Natan Portella (IDTNP), Teresina, Piauí, Brazil; Rajendra Memorial Research Institute of Medical Sciences, INDIA

## Abstract

Visceral Leishmaniasis and HIV-AIDS coinfection (VL/HIV) is considered a life-threatening pathology when undiagnosed and untreated, due to the immunosuppression caused by both diseases. Serological tests largely used for the VL diagnosis include the direct agglutination test (DAT), ELISA and immunochromatographic (ICT) assays. For VL diagnosis in HIV infections, different studies have shown that the use of the DAT assay facilitates the VL diagnosis in co-infected patients, since the performance of the most widely used ELISA and ICT tests, based on the recombinant protein rK39, are much less efficient in HIV co-infections. In this scenario, alternative recombinant antigens may help the development of new serological diagnostic methods which may improve the VL diagnosis for the co-infection cases. This work aimed to evaluate the use of the recombinant Lci2 antigen, related to, but antigenically more diverse than rK39, for VL diagnosis in co-infected sera through ELISA assays. A direct comparison between recombinant Lci2 and rK39 was thus carried out. The two proteins were first tested using indirect ELISA with sera from VL afflicted individuals and healthy controls, with similar performances. They were then tested with two different sets of VL/HIV co-infected cases and a significant drop in performance, for one of these groups, was observed for rK39 (32% sensitivity), but not for Lci2 (98% sensitivity). In fact, an almost perfect agreement (Kappa: 0.93) between the Lci2 ELISA and DAT was observed for the coinfected VL/HIV patients. Lci2 then has the potential to be used as a new tool for the VL diagnosis of VL/HIV co-infections.

## Introduction

Visceral Leishmaniasis (VL) is considered a critical and opportunistic infection in people with HIV-AIDS [1]. This is due to the action of the HIV virus, which reduces the number of TCD4 + lymphocytes and facilitates the development of VL, and to interactions of the protozoan *Leishmania infantum* with the immune system, favoring virus replication and the progression of the HIV infection to AIDS [2]. The co-infection can be fatal when undiagnosed and untreated, due to the immunosuppression caused by both diseases. Even when properly treated the mortality rate is high and can reach up to 20% or more [3–5].

The visualization of the *Leishmania* parasite in bone marrow aspirates is the gold standard test for the VL diagnosis. It is highly specific, despite a low sensitivity, but it is an invasive method that can pose risks to the patients, especially those infected with HIV-AIDS [6–8]. Spleen aspirates have shown greater sensitivity but carry the risk of serious bleeding [2, 9, 10]. Serological methods are largely used as an alternative for VL diagnosis. This is mainly due to the easier sample collection, as well as to the various diagnostic methods available: immunofluorescence, ELISA, direct agglutination test (DAT) and immunochromatographic tests (ICT). These methods vary in sensitivity and specificity, since they depend on the antigens used in the test as well as on the humoral response of the patients, also a consequence of the infection prevalence and the *Leishmania* species involved [2, 11, 12]. Although the serological tests do not differentiate between active infection and cured VL, they remain as viable choices for the screening of VL/HIV co-infected samples, requiring simple procedures and less specialized equipment [4–6].

Although VL is usually associated with higher titers of antibodies against *Leishmania* proteins, facilitating the serological diagnosis, the repression of the immune system by the HIV virus reduces these significantly in co-infected patients [2]. In this scenario, different studies have shown that the use of the DAT assay facilitates the VL diagnosis in co-infected patients [13, 14]. DAT is a semi-quantitative serological test based on the incubation of different dilutions of sera with a lyophilized extract of whole *L*. *donovani* promastigotes, having a sensitivity for VL patients of roughly 94%, with ~97% specificity [11, 14]. The DAT sensitivity in VL/HIV co-infected patients ranges between 80 to 90%, with ~90% specificity, confirming it to be an efficient test for the screening of the co-infection [2, 14]. Major disadvantages, however, are the need for multiple pipetting, relatively long incubation times and high acquisition costs, presumably due to the limited production of good quality antigens. False-positive results were also observed in patients infected with closely related protozoan parasites, such as *Trypanosoma* and other *Leishmania* species [15, 16].

Recombinant antigens have been routinely used for VL diagnosis as part of serological tests based on ELISA and ICT assays, some of which are commercially produced and with high sensitivities and specificities [6, 17]. Indeed, recent studies evaluating commercial tests available for human VL diagnosis, generally based on the recombinant rK39 antigen, have found sensitivity values ranging from ~80 to ~96%, with specificities between ~95 to 100%. Equivalent tests, however, are much less efficient with samples from HIV co-infections, with the sensibility for VL diagnosis being lower than 70% [14, 16, 18]. In this scenario, alternative recombinant antigens may help the development of new serological methods which may improve the VL diagnosis for the VL/HIV co-infections.

Different recombinant antigens potentially useful for human VL diagnosis were previously reported by some of us and which were identified through the serological screening of an *L*. *infantum* cDNA library. When tested with sera from VL-infected patients using an ELISA assay, the best recombinant antigen, Lci2, displayed 97% sensitivity and 96% specificity [19]. Lci2 and rK39 are distinct fragments of the same *L*. *infantum* protein, a kinesin, having in common the presence of several sets of similar 39 amino acid repeats. None of the repeats found within Lci2, however, is 100% identical to the repeats found within rK39, and the Lci2 repeats are more diverse in sequence [19]. In this study, we evaluated the potential for the recombinant Lci2 to confirm the VL diagnosis in VL/HIV patients through ELISA assays. When compared with a rK39-based assay, we found a substantial improvement using the Lci2 antigen.

## Materials and methods

### Serum samples

Patients were recruited from hospitals based on the Brazilian States of Piaui and Pernambuco. All human sera were collected after approval of their use by the appropriate ethics committees from public services specializing in infectious diseases. Human sera samples were divided into five groups: VL—49 sera from patients with parasitologically confirmed VL diagnosis; VL/HIV-A—16 HIV sera with parasitologically confirmed VL diagnosis; VL/HIV-B—47 HIV sera with the VL diagnosis confirmed by DAT assays; Control—50 sera from healthy control individuals, all negative for VL and HIV; HIV—12 HIV positive sera with a VL negative diagnosis confirmed through DAT assays. All HIV patients were using Highly Active Antiretroviral Therapy (HAART) and sera samples from individuals under treatment or afflicted by pulmonary tuberculosis or leprosy were not included.

### Expression and purification of the Lci2 recombinant protein

His-tagged recombinant Lci2 (originally named Lci2B) was expressed in *Escherichia coli* BL21(DE3) pLysS (Invitrogen) and affinity purified with Ni-NTA Agarose beads (Qiagen) as previously described [19]. Briefly, the transformed bacterial cells were first grown in 500 mL batches of Luria-Bertani medium supplemented with 100 μg/mL ampicillin at 37 °C until reaching a 600 nm OD of 0.5. Expression of recombinant Lci2 was induced with 0.2 mM IPTG at 30 °C for 4 h. Bacterial cells were harvested by centrifugation followed by resuspension in 20 mL of denaturing lysis buffer (100 mM sodium phosphate, 10 mM Tris, 8 M Urea, 20 mM imidazole, pH 8.0) and membrane disruption through sonication on ice. For purification, 400 mL of the Ni-NTA beads was added, followed by washes in lysis buffer with 50 mM imidazole (pH 6.0) and elution in the same buffer with 1 M imidazole (pH 4.5), all under denaturing conditions. Purified protein products were analyzed by 15% SDS-PAGE gels stained with Coomassie blue R-250. To determine the recombinant protein concentration, densities of the Lci2 bands in Coomassie blue-stained gels were compared with those of known concentrations of BSA.

### ELISA

For Lci2, the ELISA was carried out essentially as previously described [19], with ~400 ηg of the recombinant protein, diluted in 50 mM carbonate-bicarbonate (Na_2_HCO_3_/NaHCO_3_) buffer, pH 9.6, added to each well of ELISA plates, followed by blocking with Phosphate-Buffered Saline plus 0.05% Tween-20 (PBS-T), pH 7.2, supplemented with 10% non-fat milk. The wells were then incubated with the selected sera, diluted in PBS-T plus 10% non-fat milk, at a dilution of 1:900. For the rK39 ELISA, the commercial recombinant rK39 antigen was purchased from Rekom Biotech (Granada, Spain) and the assays carried out also as described [20, 21], with ~400 ηg of rK39 added per well, diluted in carbonate-bicarbonate buffer, and blocked with PBS-T plus 3% BSA. The sampled sera were diluted in PBS-T supplemented with 4% Fetal Bovine Serum, for a final dilution of 1:50 per well. For both ELISA assays, the incubation with the secondary antibody was carried out using a peroxidase-conjugated goat anti-human IgG, diluted 1:10000. The optical density reading was performed at 490 nm in the Benchmark Plus Microplate Manager 5.2 (BIO-RAD) ELISA reader.

### Direct agglutination test (DAT)

Several dilutions of the sera were added in microtiter plates to 50 μg of lyophilized and stained crude extract of *Leishmania* promastigotes, from the *L*. *donovani* complex. The assays were carried out following the manufacturer’s specifications (Royal Tropical Institute, Amsterdam, The Netherlands), with the agglutination observed after overnight incubation. Titers ≥ 1:6,400 [22] were confirmatory for VL diagnosis.

### Statistical analyses

Sensitivity, specificity, accuracy, and confidence interval parameters were estimated using the MedCalc website. Scatterplots, ROC curve, and AUC were generated using GraphPad Prism 8.0.2. The Kappa test was used to compare the DAT, parasitological test and the ELISA (rK39 and Lci2) and to calculate the agreement between DAT and the ELISA for each protein. All conclusions were drawn at the 0.05 significance level.

### Ethics statement

All human sera were collected after approval of their use by the appropriate ethics committees from the Federal University of Piaui (0116/2005) and from the Instituto Aggeu Magalhães (0121.0.095.000–08 and 13197313.6.0000.5190).

## Results

### Comparative Lci2 and rK39 performance for VL diagnosis

**Fig 1:**
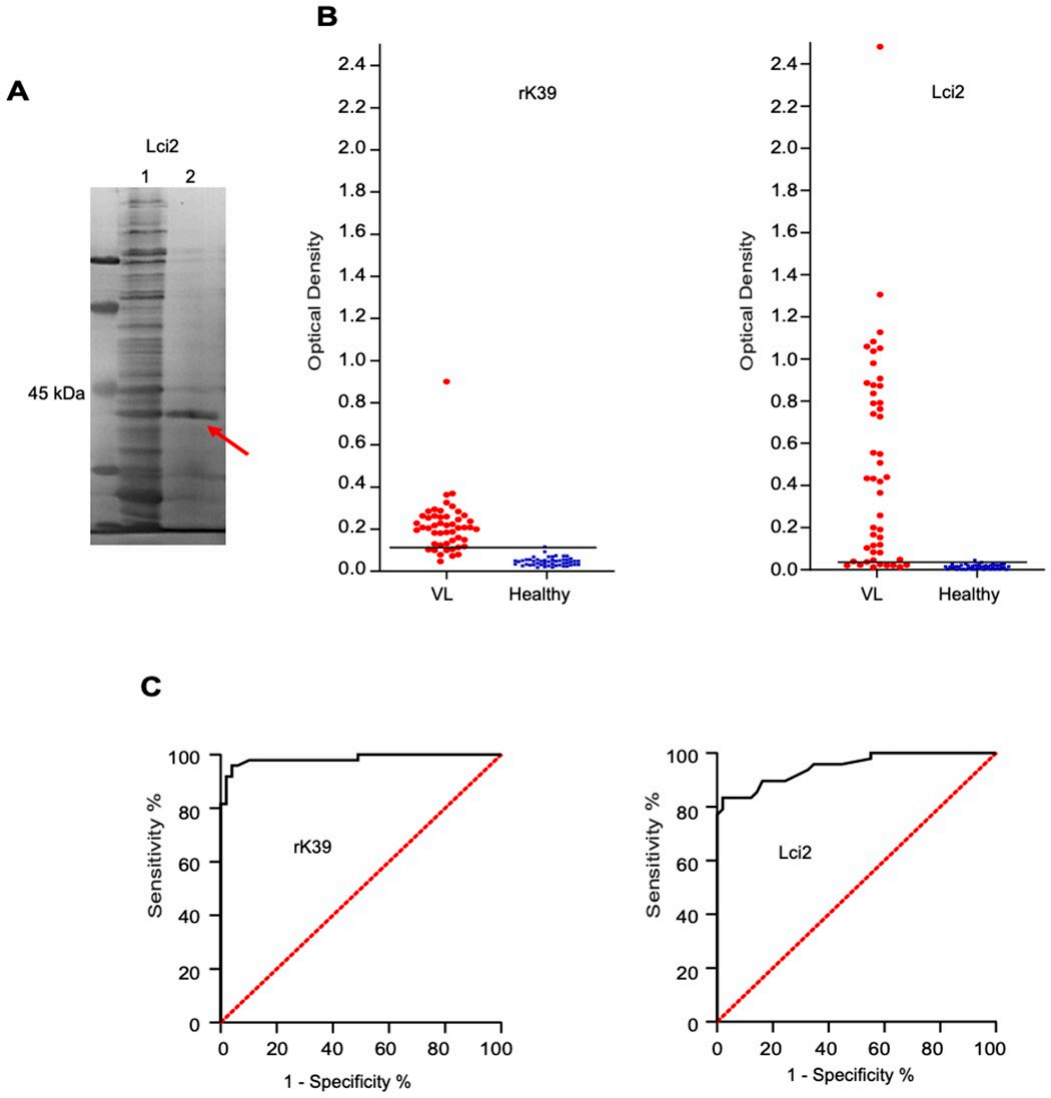
Serological evaluation of the rK39 and Lci2 recombinant antigens with sera from Visceral Leishmaniasis positive samples. **(A)** Polyacrylamide gel electrophoresis showing the Lci2 antigen evaluated in this study. **(B)** ELISA comparing the recognition of the rK39 and Lci2 recombinant proteins by human sera from VL afflicted and control individuals. **(C)** Corresponding ROC curves.

As described previously [19], the commercial rK39 and the Lci2 recombinant antigens are similarly sized polypeptides, derived respectively from the N-terminal and C-terminal regions of the same *L*. *infantum* protein, an N-terminal kinesin belonging to the kinesin-3 family (Accession in TryTripDB: LINF_140017300). These two recombinant proteins were never directly compared, so in this study we decided first to evaluate both regarding their efficiency for VL diagnosis, using a commercially purchased rK39 and a newly prepared batch of recombinant Lci2 (Fig 1A). ELISA assays were then performed testing the two proteins with the same set of 49 sera from VL-positive/HIV-negative individuals and 50 healthy controls, with the results shown in Fig 1B. Overall, similar profiles were observed for both proteins, although the optical density readings for Lci2 were higher and much more variable than for the commercial rK39. Positivity was established based on the average of the readings from the healthy control sera plus two standard deviations (95% confidence). As summarized in Table 1, values of 83% sensitivity and 96% specificity were observed for Lci2, while rK39 had a profile of 92% sensitivity and 96% specificity and accuracy values all greater than 85%. DAT assays were also carried out with the same sets of sera, resulting in higher sensitivity (96%) and specificity (100%) than either of the two recombinant proteins, when compared with heathy controls. ROC curves were also generated to compare the performance of the two ELISA tests (Fig 1C). Despite having an area under the ROC curve above 0.900, indicating a very good performance for VL diagnosis, Lci2 still had a performance slightly inferior to rK39 with the conditions assayed. However, using the conditions previously defined for each assay (see Methods), the Lci2 ELISA used the sera diluted 1:900 while for rK39 they were diluted only 1:50. This 18-fold difference in sera dilution, with minor differences in performance, indicates a likely greater potential for Lci2 for VL diagnosis using ELISA, when compared with rK39.

**Table 1:** Comparative analysis of the diagnostic performance of the ELISA assays based on the rK39 and Lci2 recombinant proteins as well as DAT. Sera from patients belonging to both VL or VL/HIV groups were evaluated.

**VL**				
**Test**	***Sensitivity (95%CI)***	***Specificity (95%CI)***	***Accuracy (95%CI)***	***Kappa index (95% CI)***
rK39 ELISA	92 (80–98%)	96 (86–99%)	94 (87–98%)	0.88 (0.78–0.97)
Lci2 ELISA	83 (70–92%)	96 (86–99%)	90 (82–95%)	0.79 (0.67–0.91)
DAT	96 (86–99%)	100 (93–100%)	98 (93–100%)	0.95 (0.93–1.00)
**VL/HIV-A**				
rK39 ELISA	75 (48%–93%)	96 (86–100%)	91 (81–96%)	0.74 (0.55–0.94)
Lci2 ELISA	94 (70–100%)	96 (86–100%)	95 (87–99%)	0.88 (0.74–1.00)
DAT	75 (47–93%)	100 (93–100%)	94 (85–98%)	0.74 (0.55–0.94)
**VL/HIV-B**				
rK39 ELISA	32% (19–47%)	96 (86–100%)	67 (54–74%)	0.28* (0.13–0.43)
Lci2 ELISA	98 (89–100%)	96 (86–100%)	97 (91–99%)	0.93 (0.86–1.00)

DAT = Direct Agglutination Test. CI = Confidence Interval. Kappa = p < 0.0001,

* p < 0.0002

### Comparative analysis for VL diagnosis in VL/HIV samples

**Fig 2:**
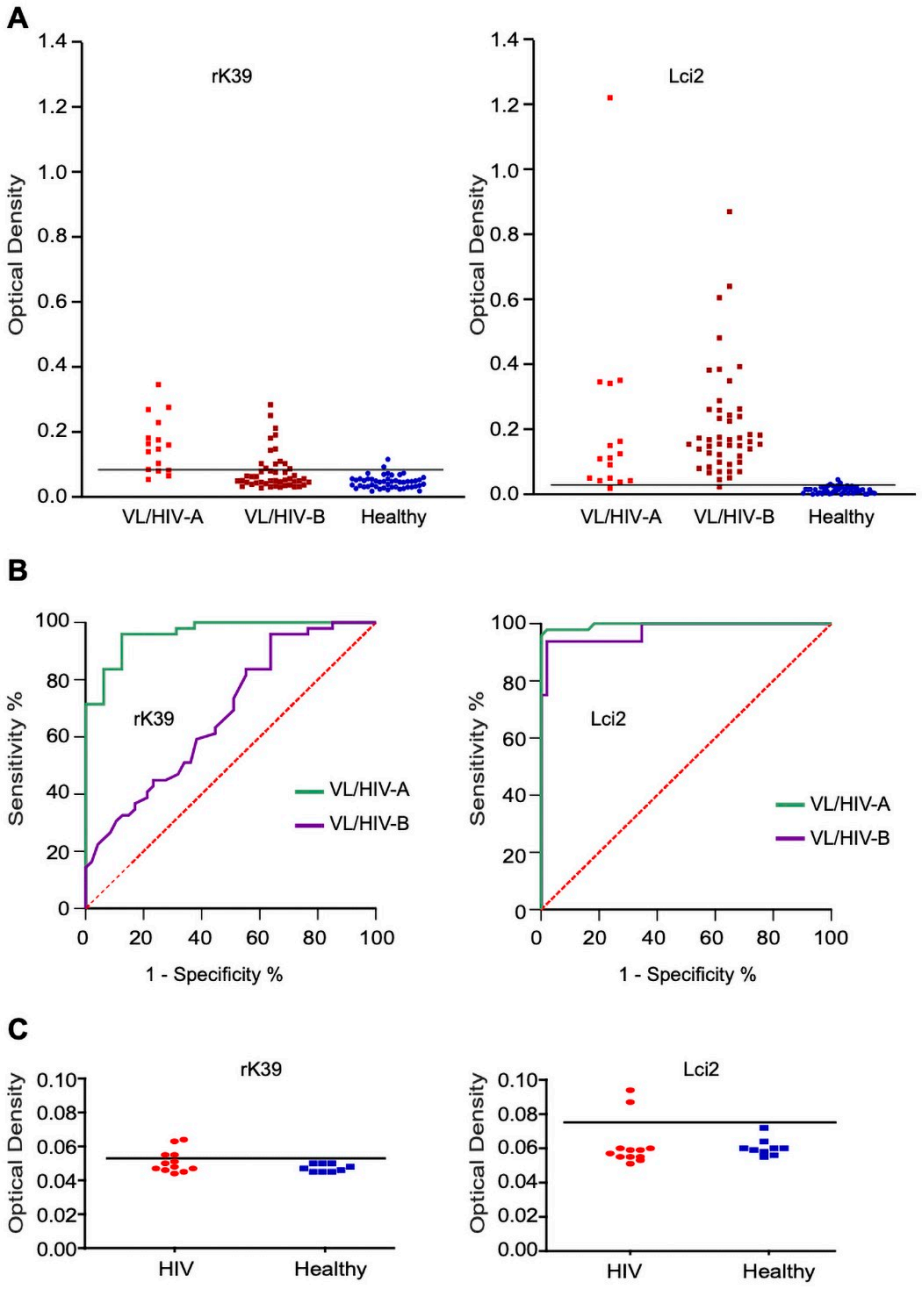
Serological evaluation of rK39 and Lci2 recombinant antigens with Visceral Leishmaniasis/HIV sera samples. **(A)** ELISA comparing the recognition of the rK39 and Lci2 recombinant proteins by human sera from VL/HIV afflicted individuals (VL/HIV-A and VL/HIV-B). **(B)** Corresponding ROC curves based on the results from the ELISA assays. **(C)** The antigens rK39 and Lci2 were also tested with sera from patients with HIV only.

Considering the poor performance previously reported for rK39 when it was used for the VL diagnosis in VL/HIV coinfections [14], we compared both Lci2 with rK39 using a group of VL/HIV sera from patients whose VL diagnosis was parasitologically confirmed (VL/HIV-A). Contrasting with the results seen for the VL-positive/HIV-negative sera, Lci2 showed a much higher sensitivity (94%) than rK39 (75%) for the VL diagnosis with the co-infected sera (Fig 2A; Table 1). These results were confirmed by a second set of ELISA assays testing a larger group of VL-HIV sera (47 versus 16 of the first group) whose VL diagnosis was confirmed by the DAT test (VL/HIV-B). Lci2 showed an even greater sensitivity (98%) and a much larger difference in comparison with rK39 (32% sensitivity), despite the latter using sera diluted 18-fold less. For both proteins, for the two sets of sera samples, the specificity was calculated based on the ELISA results with the sera from 50 healthy individuals and found to be the same (96%). ROC curves were then generated for both groups of sera for the two proteins (Fig 2B). Lci2 shows little variation in curve linearity between the two groups, with AUC values close to 1.0. A substantially less effective performance is observed for rK39, specially for the second group of VL/HIV co-infected sera. The Lci2 and rK39 antigens were also tested with sera from patients with HIV only (previously tested negative with DAT) to evaluate the incidence of false positive results in the presence of the HIV. Very low OD values were seen for all the sera tested for both proteins. Nevertheless, out of twelve sera tested, four (33.3%) for the rK39-based assay and two (16.7%) for Lci2 yielded OD values slightly above the cut-offline and which could be interpreted as a false positive result (Fig 2C). Overall, these results confirmed the previous reports concerning the inefficiency of rK39 for the VL diagnosis in co-infection cases, contrasting with a much better performance for Lci2.

### ELISA agreement with DAT in the diagnosis of VL/HIV coinfections

The possibility of having DAT results for the co-infected samples allowed us to compare the DAT performance with the Lci2 and rK39 serological tests, in order to evaluate the agreement between the different sets of results. First, we assessed the VL diagnosis with DAT of the parasitologically confirmed VL sera from the VL/HIV-A group. Remarkably the overall performance in sensitivity for DAT (75%) was identical to that seen for the rK39 ELISA and inferior to that of the Lci2 ELISA (Table 1). When considering the agreement between these results, comparing the serological tests used with the sera screened by the parasitological test in the VL group, we can observe an almost perfect agreement between DAT (Kappa: 0.95) and rK39 (Kappa: 0.88) and a substantial agreement between DAT and Lci2 (Kappa: 0.79). In contrast, in the VL/HIV-A group, we have an almost perfect agreement between DAT and Lci2 (Kappa: 0.88) but a substantial agreement between DAT and rK39 (Kappa: 0.74 for both). For the VL/HIV-B group, with all the sera included having a positive DAT result (100%), Lci2 shows an almost perfect agreement with DAT (Kappa: 0.93) while rK39 shows only a fair agreement (Kappa: 0.28), as shown in Table 1. Again, these results reinforce the very efficient performance, even when compared with DAT, of the ELISA based on the Lci2 antigen.

## Discussion

A serological test for VL diagnosis should ideally produce an early, conclusive, simple and economical diagnosis, constituting an important tool for preventing the evolution of the disease, reducing its severity and lethality. Recombinant proteins can be easy and low-cost to produce, and the assays based on these proteins, especially the ICT tests, do not require special training or equipment and can be applied in the field to produce fast results. The choice of the antigen to be used then is the most limiting factor and it is known that proteins with tandem repeat domains, having two or more copies of a specific sequence motif, are often strong B-cell stimulators, potentially able to induce the production of high antibody titers. The proteomes of the *Leishmania* and *Trypanosoma* parasites are known to be enriched with proteins having in tandem repeat domains, some of which with a large number of repeats. It is believed that this favors a strong antibody response by the host to these proteins, a response that might be stronger as the repeat copy number increases [23]. Reports in the literature confirm that proteins having large numbers of tandem repeats can alone promote an antibody-specific immune response triggered by B cells. These proteins have been repeatedly identified in serological screenings for antigenic proteins from different parasitic protozoans [24]. Indeed both rK39 and Lci2 have been identified through the screening of expression libraries with sera from VL-afflicted individuals [19, 25]. However, VL can be caused by distinct *Leishmania* parasites, and the immune response by the hosts may depend on their genetic backgrounds, leading to potentially very different responses [26]. Nevertheless, our results reinforce the Lci2 potential to be used as an efficient alternative for VL diagnosis.

The DAT is a simple, reliable and low-cost test for diagnosis of VL alone and in co-infection with HIV it is generally used because of its good sensitivity and specificity in human populations with and without the virus [14, 16, 27]. However, the DAT preparation requires culturing live parasites to obtain antigens and difficulties in the standardization of this step may lead to variation in the test results [15]. Other antigens derived from killed *L*. *infantum* or *L*. *donovani* promastigotes could likewise increase false-positive results in patients infected with other parasites, specially more closely related ones, such as other *Leishmania* species causing cutaneous leishmaniasis or even *Trypanosoma* species. Recombinant proteins would be more adequate, especially those based on native antigens whose sequences diverge even between phylogenetically related species. Indeed, only 50 to 60% identity in sequence is generally seen for the *L*. *infantum* Lci2 segment when it is compared with equivalent segments from orthologues found in other *Leishmania* species and little or no sequence identity is seen with candidate *Trypanosoma* orthologues. This is in agreement with our previous study seen very little false positive results for Lci2 using sera from patients with cutaneous leishmaniasis or Chagas Disease [19]. Considering the lower efficiency of the rK39 antigen, the data available so far clearly highlights the potential for the Lci2 to be an alternative to DAT for VL diagnosis in HIV-coinfected cases.

The higher sensitivity values of Lci2 compared to rK39 in this study for the VL/HIV coinfection groups may be explained due to the more variable sequences of the Lci2 repeats, in comparison with rK39 [19]. Regarding the VL/HIV samples, it is important to note that the sensitivity and specificity results seen for Lci2 are comparable to other studies with recombinant antigens previously evaluated for the serological diagnosis of human VL [27] and are innovative to VL/HIV coinfection. As for study limitations, the majority of our samples for VL/HIV groups were selected based on a previous positive result with DAT, since the gold-standard parasitological test carries the risk of serious bleeding in HIV patients. Our study highlights then a novel recombinant protein that could be used for the diagnosis of VL/HIV patients. More studies and testing are required for its validation, but this study represents a first step of the wider evaluation required for Lci2.
